# A perforated microhole-based microfluidic device for improving sprouting angiogenesis *in vitro*

**DOI:** 10.1063/1.4994599

**Published:** 2017-10-10

**Authors:** Sijia Chen, Liguang Zhang, Yi Zhao, Ming Ke, Bo Li, Longcong Chen, Shaoxi Cai

**Affiliations:** 1 Key Laboratory of Biorheological Science and Technology of the State Ministry of Education, College of Bioengineering, Chongqing University, Chongqing 400044, China; 2 School of Education, Chongqing Normal University, Chongqing 401331, China; 3 Medical Information College, Chongqing Medical University, Chongqing 400016, China

## Abstract

Microfluidic technology is an important research tool for investigating angiogenesis *in vitro*. Here, we fabricated a polydimethylsiloxane (PDMS) microfluidic device with five cross-shaped chambers using a coverslip molding method. Then, the perforated PDMS microhole arrays prepared by soft lithography were assembled in the device as barriers; a single microhole had a diameter of 100 *μ*m. After injecting type I collagen into the middle gel chamber, we added a culture medium containing a vascular endothelial growth factor (VEGF) into the middle chamber. It would generate a linear concentration gradient of VEGF across the gel region from the middle chamber to the four peripheral chambers. Human umbilical vein endothelial cells (HUVECs) were then seeded on the microhole barrier. With VEGF stimulation, cells migrated along the inner walls of the microholes, formed annularly distributed cell clusters at the gel-barrier interface, and then three-dimensionally (3D) sprouted into the collagen scaffold. After 4 days of culture, we quantitatively analyzed the sprouting morphogenesis. HUVECs cultured on the microhole barrier had longer sprouts than HUVECs cultured without the barrier (controls). Furthermore, the initial distribution of sprouts was more regular and more connections of tube-like structures were generated when the microhole barrier was used. This study introduces a novel microfluidic device containing both microtopographic structures and 3D collagen. HUVECs cultured with the microhole barrier could form well-interconnected tube-like structures and are thus an ideal *in vitro* angiogenesis model.

## I. INTRODUCTION

Angiogenesis is the physiological process of growing new microvessels from pre-existing vessels, and it plays an important role in regenerative repair, tumor growth, and numerous other angiogenesis-dependent diseases.^1–4^ The critical steps during new blood vessel formation are vascular basement membrane degradation, directional migration of endothelial cells (ECs), sprouting morphogenesis, and the formation of tube-like structures.^5^ Since the first *in vitro* angiogenesis model and the hypothesis that solid tumors are angiogenesis dependent were presented by Folkman,^6,7^ the study of angiogenesis-dependent diseases has become a hotspot.^8–10^ The development of a suitable *in vitro* angiogenesis model has thus become a key step to understanding the cellular and molecular mechanisms of angiogenesis.

Recently developed microfluidic technology, which is based on microelectromechanical systems (MEMS) technology, has gradually become an important method for establishing *in vitro* angiogenesis models.^11–14^ The microfluidic technologies make it possible to better control studies of the effects of physical and chemical factors on angiogenesis in three dimensions (3D). The various existing microfluidic devices have allowed for the construction of microvascular networks,^13,15^ 3D co-culture of ECs and angiogenesis-related cells,^14,16^ the establishment of controllable concentration gradients of angiogenesis-related factors (such as vascular endothelial growth factor, VEGF),^12,13^ investigations of the effect of extracellular matrix (ECM) biophysical and biochemical properties on angiogenesis,^17^ investigations of the effect of mechanical stimulation on angiogenesis,^18,19^ and other experiments. However, the shapes of the vertical interfaces between cell chambers (or channels) and gel chambers (or channels) in these microfluidic devices were mostly rectangular^11–14,18^ (the cell chamber here refers to the chamber in which cells are seeded and in some studies also known as the media chamber). Considering that the basic structure of blood vessels is a circular tube-like structure with a lumen, it may be more appropriate to construct circular structures on the cell-gel chamber (or channels) interfaces to replace the common rectangular structures. Obviously, this will be closer to the real situation of vascular sprouting.^20,21^

In recent years, topographic substrates have been widely used in cell culture and relevant researches.^22–25^ These substrates are on a micro- or nanoscale and could significantly affect the cell morphology, adhesion, migration, and distribution,^24–26^ and thus, they could also affect cell proliferation, differentiation, and function.^24,25^ Our previous study indicated that cells seeded on a microwell substrate with cylindrical sidewalls would grow along the circumferential direction of the sidewalls.^27^ In addition, most *in vivo* ECs grow on the inner wall of blood vessels with a variety of cylindrical concave surfaces. In view of this, we speculated that the use of a microhole with a cylindrical concave surface may also direct ECs into an annular distribution,^27,28^ thus improving sprouting morphogenesis in a 3D collagen scaffold and providing an ideal *in vitro* angiogenesis model.^20,29^ Additionally, this would also be a new attempt and exploration of the integration of microtopographic substrates and 3D matrices for cell culture.

In this study, we fabricated a novel polydimethylsiloxane (PDMS) microfluidic device with a perforated PDMS microhole barrier. A coverslip molding method and soft lithography were used to fabricate the main device and the microhole barrier. Type I collagen, a major protein of the ECM, was injected into the gel chamber of the microfluidic device to serve as the scaffold and establish a 3D microenvironment.^30^ Then, we added the medium supplemented with VEGF into the gel chamber to induce the directional sprouting of human umbilical vein endothelial cells (HUVECs) into the collagen scaffold.^31^ Fluorescein isothiocyanate (FITC)-dextran and the finite element method (FEM) were used to investigate the diffusion profile of VEGF in the collagen scaffold.^12,32^ Finally, we compared the differences in sprouting morphogenesis between HUVECs cultured with and without the barrier.

## II. MATERIALS AND METHODS

### A. Microfluidic device fabrication

**FIG. 1.**
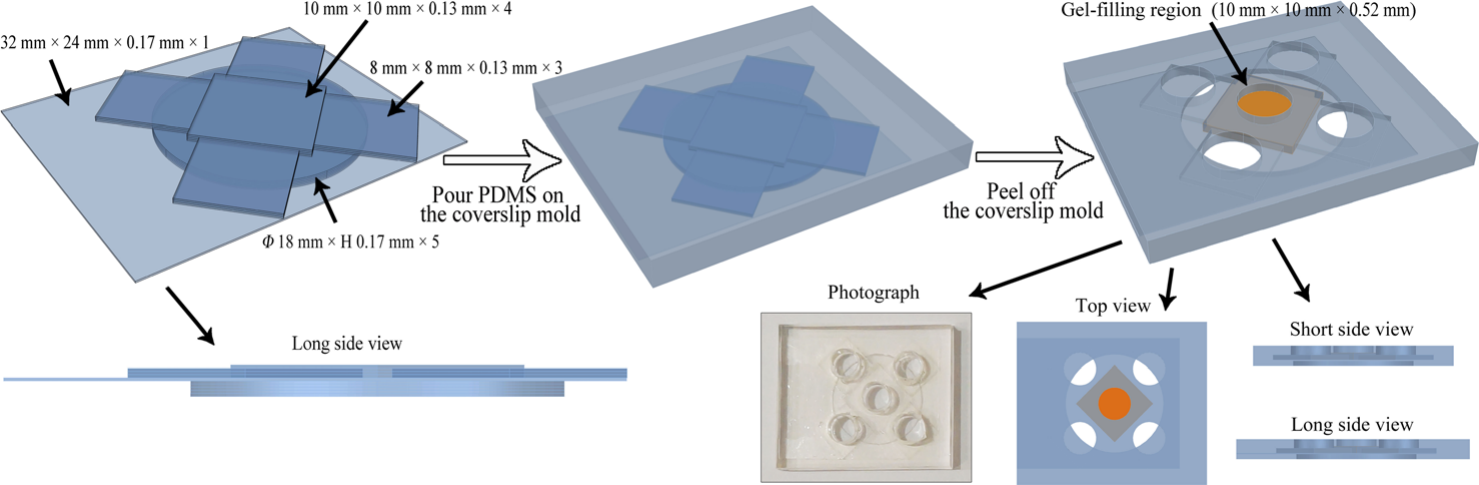
Schematic illustration of the coverslip molding method for the fabrication of the PDMS microfluidic device. The left schematic shows the arranged coverslips. The right schematic shows the top view, short side view, and long side view of the final device. The middle gel chamber is marked in orange. The lower middle photograph shows the fabricated device.

We fabricated a PDMS microfluidic device using a coverslip molding method (Fig. 1). It was composed of one middle gel chamber (W × L × H, 10 mm × 10 mm × 0.52 mm) and four peripheral cell chambers (each chamber: W × L × H, 8 mm × 8 mm × 0.39 mm). A circular window (*Φ*, 18 mm; H, 0.85 mm) was created at the bottom for subsequent experimental observations. Using PDMS and standard coverslips, we fabricated the microfluidic device as follows: (1) Coverslips of different sizes were arranged in a Petri dish from bottom to top: five circular coverslips (*Φ*, 18 mm), one rectangular coverslip (32 mm × 24 mm), and four square coverslips (10 mm × 10 mm) were placed in the center, and four groups of three square coverslips (8 mm × 8 mm) were placed around. The thickness of the square coverslips was 0.13 mm, and all coverslips were glued together with cyanoacrylate glue. (2) The PDMS (Sylgard 184, Dow Corning, USA) precursor and curing agent were mixed in a mass ratio of 10:1, vacuum degassed, poured carefully over the Petri dish containing the assembled coverslips, and cured at 60 °C for 3 h. (3) After cooling, the PDMS device was peeled off from the Petri dish and cut into the appropriate size. All coverslips were carefully removed, and then, we punched five 6-mm diameter holes in each chamber as reservoirs.

### B. Microhole barrier fabrication

**FIG. 2.**
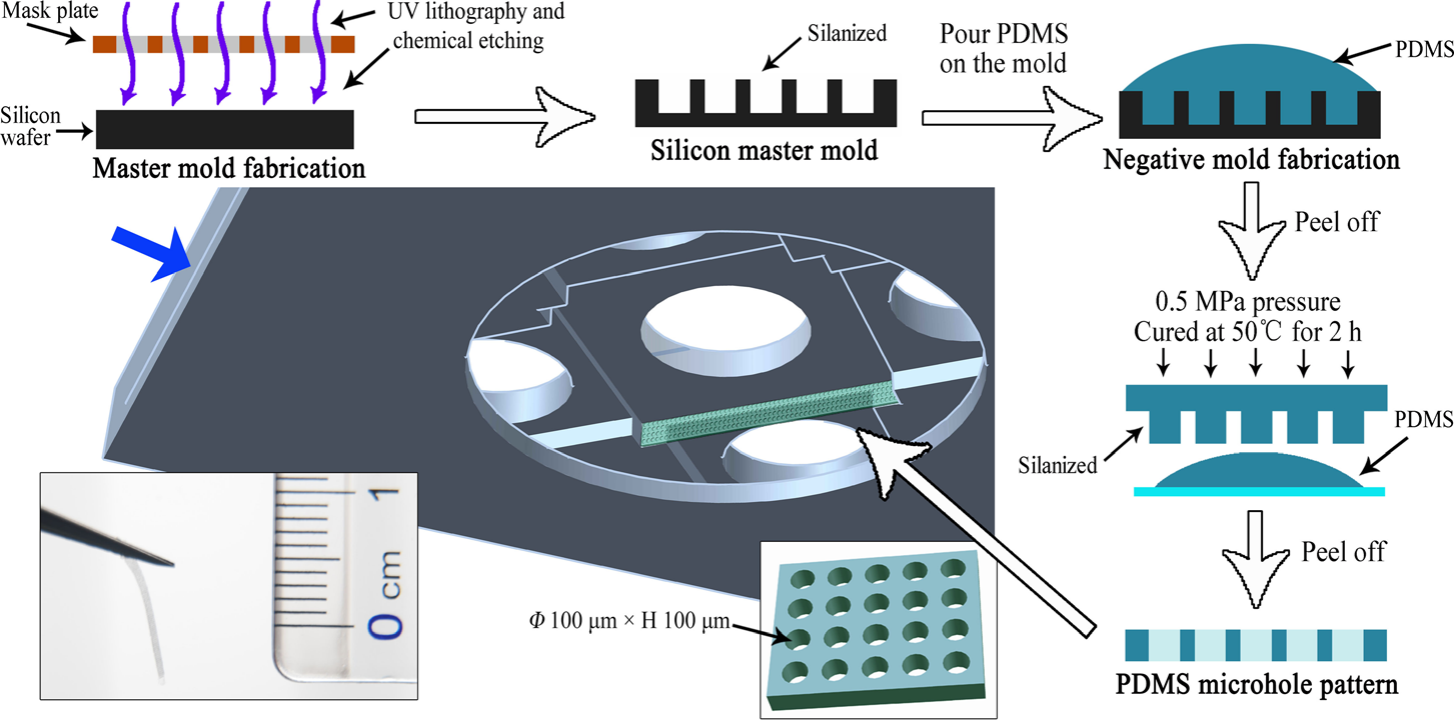
Schematic illustration of the protocol used to fabricate the PDMS microhole barrier. The microhole barrier was fabricated by soft lithography as shown on the top and right sides of the schematic. The middle schematic shows the location of one barrier, and the lower left photograph shows the fabricated PDMS microhole barrier. The blue arrow indicates the insertion position of the coverslip before each experiment.

The barrier was composed of a perforated PDMS microhole array, with each microhole having a diameter of 100 *μ*m and a height of 100 *μ*m. The silicon master mold (p-type ⟨100⟩ silicon wafer, GRINM, China) was prepared with a quartz-chrome mask by UV lithography. Briefly, it was fabricated as follows: thermal oxidation of the silicon, photoresist coating, UV curing, wet etching in hydrofluoric acid, and BOSCH process-based dry etching (Fig. 2). Then, the surface of the silicon master mold was silanized with 97% Trichloro (1H, 1H, 2H, 2H-perfluorooctyl) silane (Sigma-Aldrich, USA) for 30 min under vacuum to make it easier to release the subsequent PDMS negative mold. The PDMS prepolymer was prepared as described above, poured over the silicon master mold, and cured at 50 °C for 2 h after vacuum degassing. After cooling, the PDMS negative mold was peeled off from the silicon master mold and silanized as previously described. Then, we poured the PDMS prepolymer onto this negative mold, and it was vacuum degassed again. The sample was cured at 50 °C for 2 h at a pressure of 0.5 MPa. Then, the perforated PDMS microhole array was carefully peeled off the PDMS negative mold after soaking it in alcohol. After cutting, the PDMS microhole barriers were vertically fixed on the symmetrical sides of the gel chamber with the PDMS prepolymer (the middle schematic of Fig. 2 shows the location of one barrier, and in this study, the gel chamber of each device contains two symmetrical barriers; the sides of the gel chamber without the barrier are used as the control). As the device could be reused, one piece of the 32 mm × 24 mm coverslip was inserted into the position indicated by the blue arrow in Fig. 2 before each experiment. The device could be autoclaved and used in experiments.

The PDMS microhole barrier was investigated by scanning electron microscopy (SEM). After drying at room temperature, the sample was sputter-coated with gold for 1 min and viewed using a scanning electron microscope (S-3400N, Hitachi, Japan) at an acceleration voltage of 20 kV.

### C. Collagen gel preparation

Type I collagen (BD Biosciences, USA) was used as a scaffold material. The pre-polymerized collagen gel solution was prepared by adding type I collagen stock solution to a mixture of 10× phosphate-buffered saline (PBS), 1 N NaOH, and tissue culture grade water (all solutions were kept on ice) to achieve the required concentration. Then, this solution was carefully injected into the middle gel chamber of the microfluidic device (kept on ice) with a cooled pipette. The microfluidic device was placed in a Petri dish and then transferred to a 37 °C incubator for 30 min to form the 3D collagen scaffold. After gelation, the cell chambers were filled with the cell culture medium and incubated for 2 h.

### D. Characterization of the diffusion process

As HUVEC sprouting in a VEGF gradient (38.2 kDa, R&D Systems, USA) would be investigated in subsequent experiments, we first characterized VEGF diffusion in the collagen scaffold. FITC-dextran (40 kDa, Invitrogen, USA) has a similar molecular weight to VEGF and was used to visualize the concentration distribution inside the gel chamber. Collagen gel (2 mg/ml) was prepared as previously described. After gelation, FITC-dextran solution was loaded into the middle gel chamber of the microfluidic device, while an equal volume of PBS was loaded into the cell chambers. The diffusion profile was observed using an inverted fluorescence microscope (DMI 6000, Leica, Germany), and time-lapse fluorescence images were taken in the intermediate region of one side of the gel chamber for 12 h. The captured fluorescence micrographs were converted to grayscale, and the fluorescence intensity was analyzed using ImageJ (http://rsbweb.nih.gov/ij/).

To further understand the concentration distribution of VEGF in the collagen scaffold, we simulated the diffusion of VEGF using a finite element model generated in COMSOL Multiphysics (COMSOL, USA). The diffusion coefficient of VEGF in the collagen scaffold^33,34^ was assumed to be 6 × 10^−11^ m^2^/s, and the diffusion coefficient of VEGF in the water^35^ was assumed to be 3.3 × 10^−10^ m^2^/s. FITC-dextran has a similar molecular weight as VEGF, and thus, it was used as the main reference for the parameter settings. In the simulation, we filled the reservoir above the gel chamber with 200 ng/ml of VEGF and then simulated its diffusion to the peripheral cell chambers within 720 min. The detailed parameters of the model are provided in supplementary material Note 1.

### E. Cell culture with the microhole barrier

HUVECs were expanded with high-glucose Dulbecco's modified Eagle's medium (DMEM) (Gibco, USA) supplemented with 10% fetal bovine serum (Gibco, USA), 100 U/ml penicillin, and 100 *μ*g/ml streptomycin (Sigma-Aldrich, USA) on culture flasks incubated in a humidified incubator at 37 °C with 5% CO_2_.

To understand the cell growth behavior in microholes, HUVECs were cultured on a horizontally placed microhole array. The PDMS microhole array was first incubated with DMEM overnight, and then, HUVEC suspensions at a density of 1 × 10^5^ cells/ml were seeded onto the microhole array. Cells were incubated in a humidified incubator at 37 °C with 5% CO_2_ for 1 day and were investigated using a phase-contrast microscope (DM750, Leica, Germany).

HUVEC suspensions at a density of 1 × 10^6^ cells/ml were seeded in the cell chambers of the microfluidic device in which the middle chamber had been filled with collagen as previously described. To encourage the cells to grow on the vertical surface of the collagen scaffold, after the cells were seeded in each cell chamber, the microfluidic device was kept in a vertical position and incubated in a 37 °C incubator for 30 min; then, the excess cell suspension was aspirated. The device was kept flat after the above steps. The DMEM-HG medium was loaded into the peripheral cell chambers (80 *μ*l each), while the medium supplemented with VEGF (200 ng/ml) was loaded into the gel chamber (53 *μ*l). To maintain the concentration gradient of VEGF, the media in the gel chamber and cell chambers were changed every 12 h. Cells were cultured for 2 days and were investigated using a phase-contrast microscope.

### F. Characterization of sprouting morphogenesis

To further investigate HUVEC sprouting morphogenesis in a VEGF gradient, we stained the cells with a cell-permeable fluorescence probe, carboxyfluorescein diacetate succinimidyl ester (CFSE, Molecular Probes, USA). HUVECs in the exponential growth phase were suspended and centrifuged. After removing the supernatant, the cells were suspended in 2 ml of 2 *μ*mol/l CFSE solution and incubated at 37 °C for 30 min. Then, the suspension was centrifuged again to remove the unused CFSE. The DMEM-HG medium supplemented with 10% fetal bovine serum was used to resuspend the cells after washing.

The CFSE-stained HUVECs were cultured in the microfluidic device with VEGF stimulation (as described in MATERIAS AND METHODS, part E). When cultured for 1–4 days, the cells in the intermediate region of each side of the gel chamber were imaged using a laser scanning confocal microscope (LSM 510 META, ZEISS, Germany) with a scanning step of 15 *μ*m.

Based on the fluorescence results, we analyzed the following data using ImageJ: (1) The number of sprouts per unit length (500 *μ*m, which is the width of one statistical region, the same below) was determined by counting the number of tip cell clusters over a length of 80 *μ*m. (2) The length of the sprouts was calculated as the length of the tip cell clusters plus the vertical distance from the proximal position of the tip cells to the boundary of collagen. (3) The projected area per unit length was obtained by counting the projected area of all the fluorescence regions per unit length. (4) The number of pores per unit length was determined by counting the number of pores with an area greater than 1000 *μ*m^2^ within 200 *μ*m from the boundary of collagen, where the pores were generated by interconnecting tube-like structures.

To better investigate HUVEC sprouting morphogenesis in the collagen scaffold, the fluorescence results were used for 3D reconstruction by Imaris software (Bitplane, Switzerland).

## III. RESULTS AND DISCUSSION

### A. Microfluidic device for 3D culture

We designed and fabricated a novel microfluidic device with one middle chamber and four peripheral chambers (Fig. 1). Before each experiment, we merely needed to insert one coverslip into the device and sterilized it. This design prevented leakage and was convenient for optical observation. The depth of the middle chamber differed from that of the peripheral chambers by one coverslip, which was approximately 130 *μ*m. It was conducive to the adhesion of the PDMS microhole barrier to the device and could prevent the gel in the middle chamber from spilling into the cell chambers.

Type I collagen has been widely used in various studies and is often used in the construction of 3D environments in microfluidic devices.^12,30^ Different concentrations of collagen have different mechanical properties,^17,36^ which significantly affect EC sprouting and the formation of microvascular-like structures.^17^ In addition, because of the large size of our device, it was necessary to select an appropriate collagen concentration to prevent collagen collapse. In a preliminary experiment, collagen solution was prepared at five concentrations (0.5, 1, 1.5, 2, and 3 mg/ml), and VEGF-induced HUVEC sprouting was investigated. After 4 days of culture, the 0.5, 1, and 1.5 mg/ml collagen scaffolds had collapsed in varying degrees, whereas the 2 and 3 mg/ml collagen scaffolds did not. Considering that the HUVECs sprouted better in the 2 mg/ml collagen, we chose this concentration for subsequent experiments.

In this study, we fabricated a novel microfluidic device using the coverslip molding method. The whole process required only coverslips and PDMS. The device was easy to clean and could be used repeatedly. In addition, the device fabricated using this method was able to meet different requirements by changing how the coverslips were arranged. The device also exhibited good compatibility, as additional structures could be easily attached as required.

### B. Characterization of the diffusion process

**FIG. 3.**
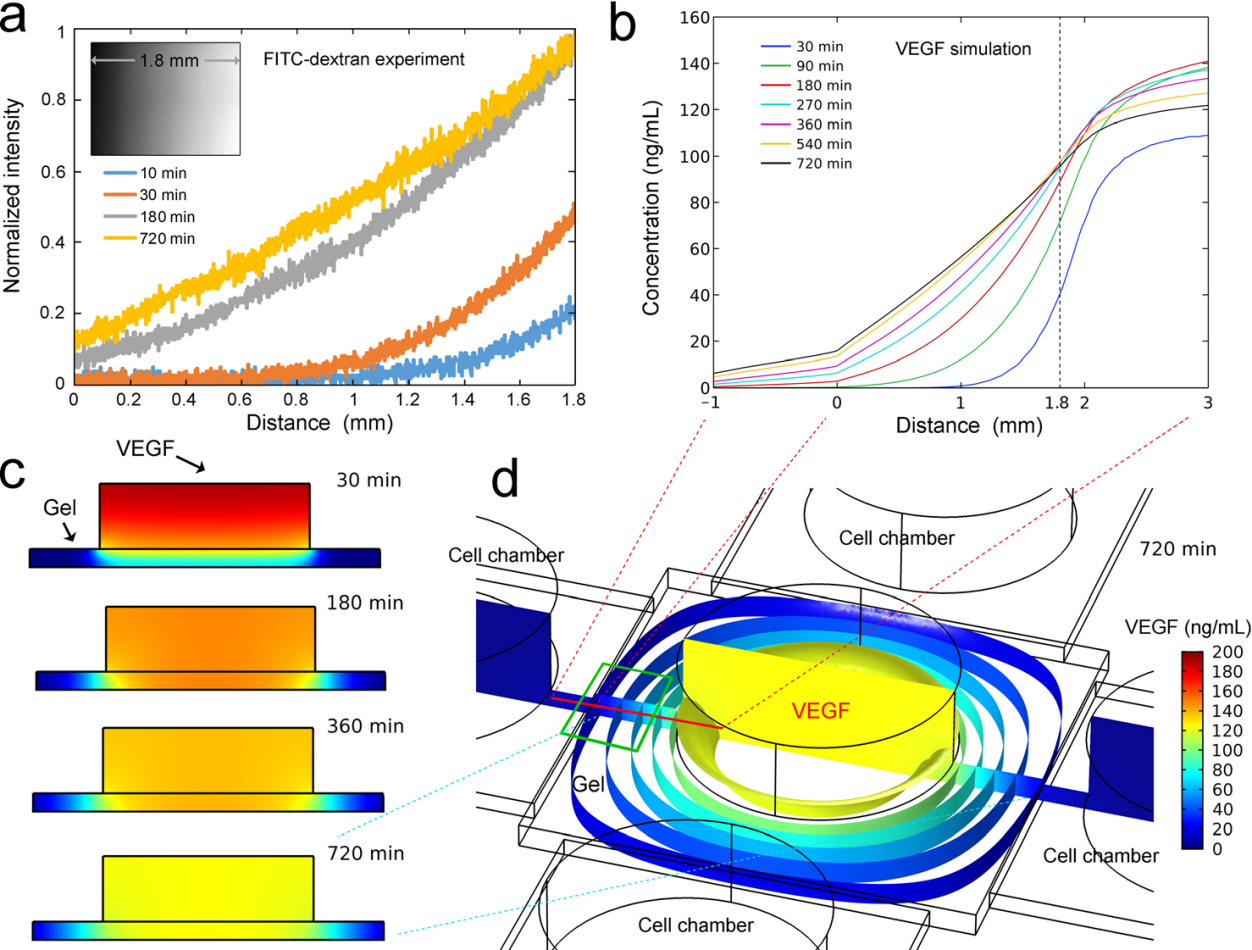
Diffusion of FITC-dextran and VEGF in the gel scaffold. (a) Diffusion of FITC-dextran in the collagen scaffold. The inset shows a grayscale representation of FITC-dextran diffusion in the gel scaffold. (b) Simulation of VEGF diffusion in the device. The position from which these data were derived is shown by the red line in (d). (c) The vertical sections showing VEGF diffusion. The position of these sections is shown by the vertical section in (d). (d) The isosurfaces and vertical section of VEGF diffusion at 720 min. The main observation region of EC sprouting is shown by the green wireframe.

Since the molecular weight of VEGF is similar to that of FITC-dextran, the concentration distribution of VEGF in the collagen scaffold was indirectly determined by analyzing the fluorescence intensity distribution of FITC-dextran.^12,17^ FITC-dextran diffused from the middle chamber to the peripheral chambers, and the fluorescence intensity in the collagen scaffold increased from the center to the periphery over time. The diffusion of FITC-dextran was analyzed in a 1.8-mm-long gel region [the actual length of one fluorescent image, see the illustration in Fig. 3(a)] from one cell-gel chamber interface to the middle chamber. The results showed that a nearly linear gradient was established within approximately 180 min. Then, the concentration gradient gradually became more linear at 720 min. Thus, to maintain a linear VEGF concentration gradient in subsequent experiments, the medium in each chamber was changed every 12 h.

Simulation of the diffusion process helped to better understand the concentration distribution of VEGF in the collagen scaffold. VEGF diffusion was simulated in the device without barriers [Fig. 3(b), simulation of the diffusion process is shown in supplementary material Video 1], and the results were consistent with the experimental result of FITC-dextran diffusion [Fig. 3(a)]. The concentration gradient was nearly linear from minute 270 to minute 720, and thus, it was appropriate to change the medium every 720 min. We also simulated VEGF diffusion in the device with the microhole barriers (Fig. S1, supplementary material), and the result was basically the same as that of the simulation using the device without the microhole barrier. Figure 3(c) shows the vertical sectional views of VEGF distribution in the middle chamber at different times. As the time increased, the concentration of VEGF in the upper region of the middle chamber gradually decreased, whereas the concentration of VEGF in the gel region gradually increased toward the periphery. From minute 180 onward, it was apparent that the concentration gradients in the peripheral gel region were basically equal at different vertical heights, which provided a uniform environment for EC sprouting. Most of the isosurfaces of diffused VEGF [Fig. 3(d)] were perpendicular to the plane of the device, which also confirmed the above conclusion. In addition, the isosurface near the edge of the gel was approximately parallel to the cell-gel chamber interface, and the isosurface became rounded toward the center of the device. To obtain a more parallel concentration profile, we investigated EC sprouting in the intermediate region of each side in the subsequent experiment [shown by the green wireframe in Fig. 3(d)].

Each chamber of the microfluidic device has a 6-mm diameter reservoir. This design helped maintain the VEGF concentration gradient for a greater duration. The concentration gradient of VEGF in collagen was maintained between 20 and 100 ng/ml, which was considered to be effective at inducing EC sprouting.^37^ We also tried to implement different concentration profiles in the device by loading VEGF into one peripheral chamber to induce EC sprouting, where cells were seeded in the middle or opposing chamber. The corresponding concentration gradient profile is shown in Fig. S2 (supplementary material). The peripheral chambers could contain up to four different chemokines in one experiment.^32^ With these concentration profiles, we were able to achieve different cell co-culture conditions. The middle chamber could also be used to investigate the behavior of cells growing vertically into the 3D matrix.^38^ All of these indicate the expansibility and functional diversity of our microfluidic device.

### C. Cell culture with the microhole barrier

**FIG. 4.**
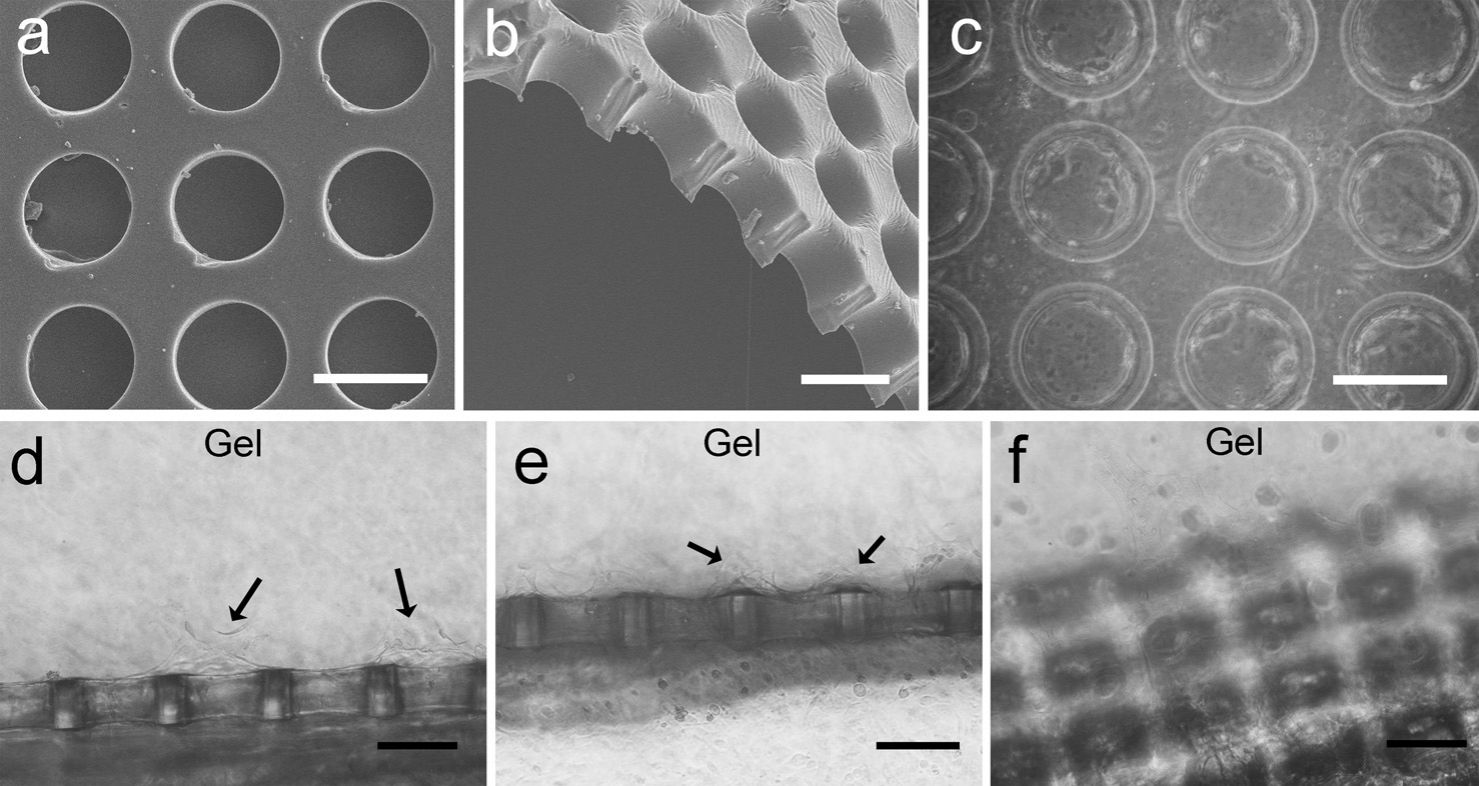
SEM images of the microhole barrier and cell integration. (a) and (b) SEM images of the PDMS microhole barrier. (b) The sectional view. (c) Phase-contrast micrograph of HUVECs cultured on the microhole substrate for 1 day. (d)–(f) Phase-contrast micrograph of HUVECs cultured in the device with the microhole barrier for 2 days. The arrows indicate the initial sprouting positions. The scale bar is 100 *μ*m.

The PDMS microhole barrier with arrayed perforated microholes was prepared by soft lithography. The front view and sectional view are shown in Figs. 4(a) and 4(b). It is clear that the microholes had been penetrated. Figure 4(c) shows HUVECs that were cultured in the PDMS microhole array for 1 day. Most of the cells had attached to the inner wall of the microhole and were distributed along the circumference. Thus, it was foreseeable that the HUVECs would be able to achieve an annular distribution at the interface of the gel and microhole barrier due to the special microtopography of the microhole.^27,28^

HUVECs were cultured in the device in the presence of a VEGF gradient. After 2 days of culture, the cells migrated out of the barrier and tended to sprout at the middle of the microhole, as shown by the arrows in Fig. 4(d). We considered that it was a natural process, and the HUVECs first annularly grew in the microhole and then gradually sprouted into the gel scaffold. However, some HUVECs sprouted between two microholes, as shown by the arrows in Fig. 4(e). It may have occurred because some cells grew along the surface of the microhole barrier instead of migrating directly into the gel scaffold. Then, after encountering cells from the adjacent microholes, they also sprouted into the gel scaffold. Compared to random EC sprouting when cultured without the barrier (data not shown), EC sprouting in the device with the microhole barrier was more regular at the initial stage. Figure 4(f) shows an oblique view of the EC growth on the microhole barrier after 2 days of culture. As shown here, there were already many cells on the barrier.

In this study, we fixed a perforated PDMS microhole barrier to the vertical plane at the junction of the middle chamber and the peripheral chamber. This arrayed microhole structure in a vertical plane could not be achieved by traditional methods of microfluidic device fabrication.^11,18^ It was also an attempt to integrate microtopographic structures and a 3D gel scaffold into a microfluidic device and take advantage of their unique properties for cell culture.^30,39,40^

### D. Characterization of the sprouting morphogenesis

**FIG. 5.**
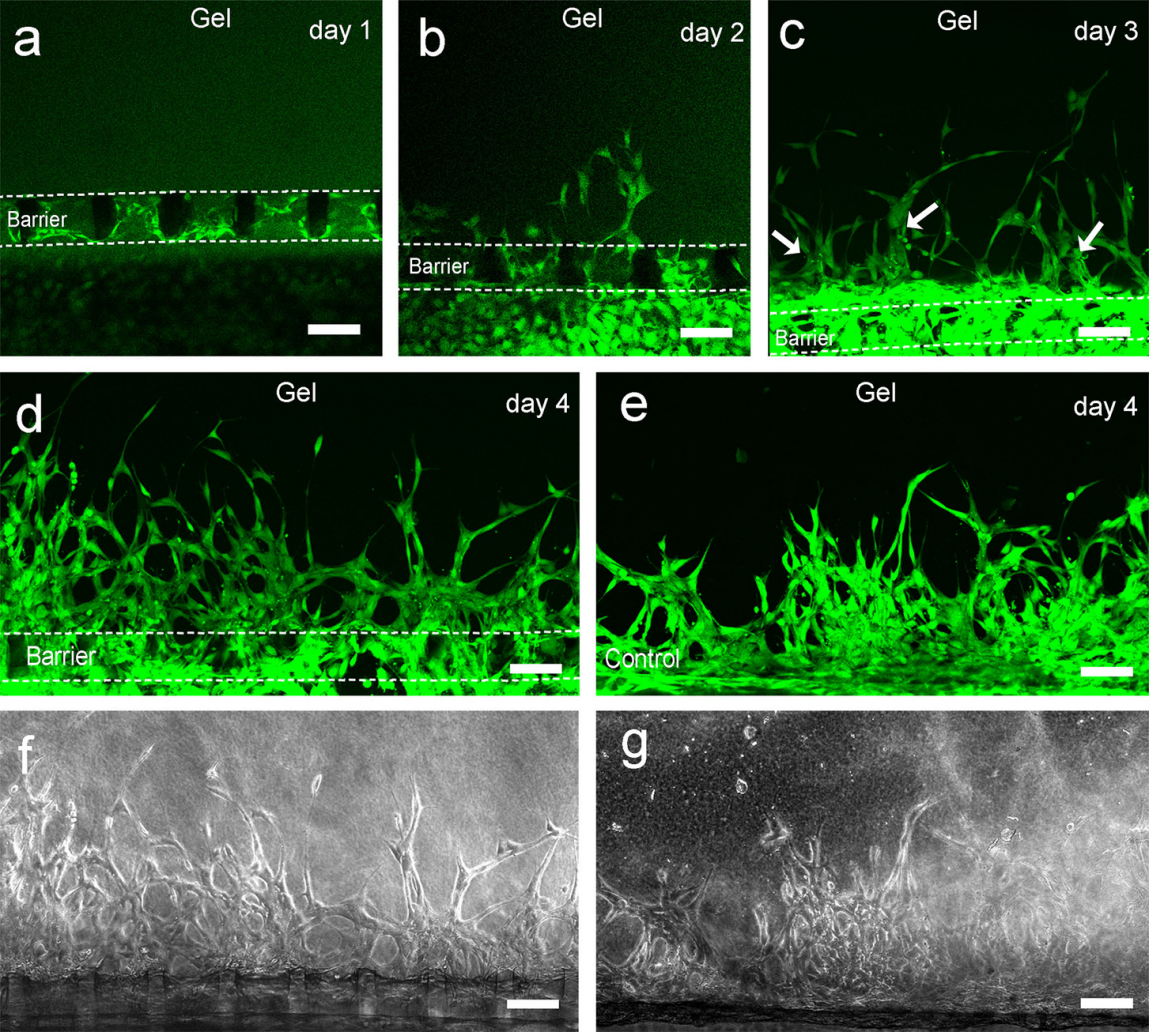
Characterization of HUVEC sprouting morphogenesis by CFSE. (a)–(d) Fluorescence micrographs of cells in the microfluidic device with the PDMS microhole barrier over a 4-day period. The edges of the microhole barrier are marked with dashed lines. The arrows in (c) indicate the tube-like structures. (e) Fluorescence micrographs of cells in the microfluidic device without the barrier after 4 days of culture. (f) and (g) Phase-contrast micrographs of (d) and (e), respectively. The scale bar is 100 *μ*m.

To comprehensively investigate the growth behavior of HUVECs, we stained the cells with the cell-permeable fluorescent dye CFSE. The preliminary experiments indicated that the cells formed sprouts faster and were more dense in the region within 100 *μ*m from the bottom of the gel scaffold. The superimposing of sprouts in multilayered microholes might cause statistical confusion, and thus, a 100–*μ*m-thick region which contained the bottom row of microholes was analyzed to circumvent this issue. After 1 day of culture, most of the microholes were covered with cells [Fig. 5(a)]. After 2 days of culture, the cells began to sprout in some of the microholes [Fig. 5(b)]. When cultured for 3 days, the cells formed many thick tube-like structures [shown with arrows in Fig. 5(c)]. In addition, as previously observed [Figs. 4(d) and 4(e)], some of these tube-like structures grew directly from the microholes, but some sprouted from the middle of two adjacent microholes. When cultured for 4 days, the cell sprouts continued to increase, and the density of sprouts increased [Figs. 5(d) and 5(f)]. The connections between the different sprouts and the tube-like structures were more abundant, and thus, many pores were formed. For a more comprehensive display of the sprouting morphogenesis after 4 days of culture, more pictures from different devices are shown in Fig. S3 (supplementary material). We also investigated the sprouting of cells cultured for 4 days without using the microhole barrier as a control [Figs. 5(e) and 5(g)]. Under this condition, the cells accumulated near the boundary of collagen, and the sprouts were more random; they intertwined with each other and formed fewer pores.

**FIG. 6.**
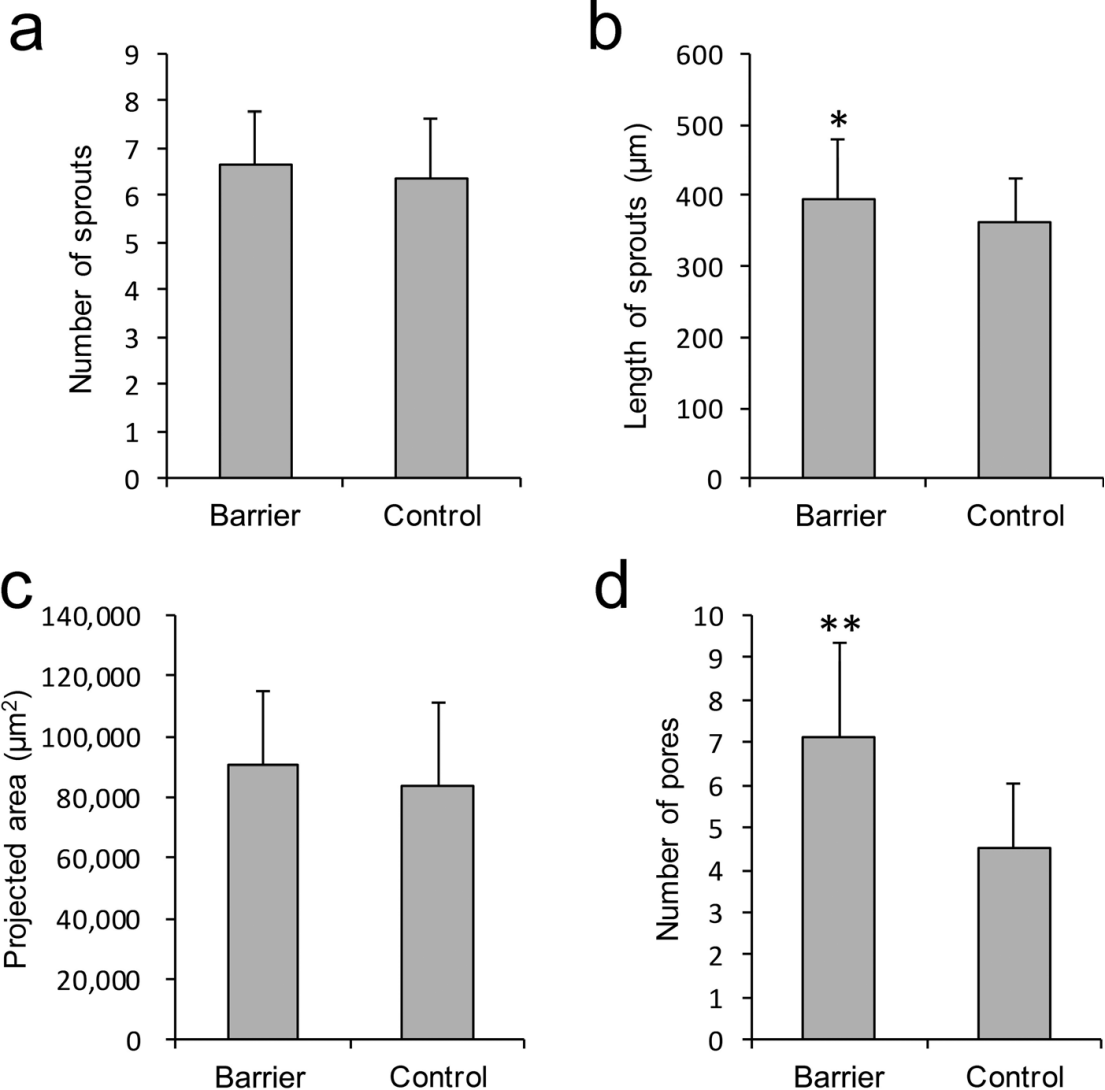
Quantitative assays of HUVEC sprouting. (a) The number of sprouts. (b) The length of sprouts. (c) The projected area of the sprouting region. (d) The number of pores formed by interconnecting sprouts. These results are based on the fluorescence data in a 500-*μ*m-long statistical region (*P < 0.05, **P < 0.001; Student's *t*-test, n = 5 devices; error bars are presented as ±SD).

To quantitatively compare the differences in HUVEC sprouting between the cells cultured in the chamber with the PDMS microhole barrier and those cultured in the control chamber, we statistically analyzed the fluorescence results after 4 days of culture. All the results are based on the data derived from a 500–*μ*m-long statistical region (detailed statistical methods are shown in Fig. S4 in the supplementary material). Figure 6(a) shows that there was no significant difference in the number of sprouts between the barrier group and the control group. The length of the sprouts in the barrier group was significantly greater than that in the control group [Fig. 6(b), *P* < 0.05], and there was no significant difference in the projected area of the sprouting region between the two groups [Fig. 6(c)]. In addition, the number of pores formed by interconnecting tube-like structures in the barrier group was significantly greater than that in the control group [Fig. 6(d), *P* < 0.001]. Our analysis of the mean fluorescence intensities with respect to different distances from the boundary of collagen also confirms the above results (Fig. S5, supplementary material).

Among the results, the number of pores was the most significantly different parameter between the barrier group and the control group. This was because the special configuration of the microhole barrier limited the region of EC sprouting.^27,28,41^ The cells thus formed more regular sprouts at the initial stage of sprouting. Due to the established spacing between the microholes, there was spacing between the sprouts. After the sprouts connected to each other, they naturally formed more interconnected tube-like structures.^13,42^

**FIG. 7.**
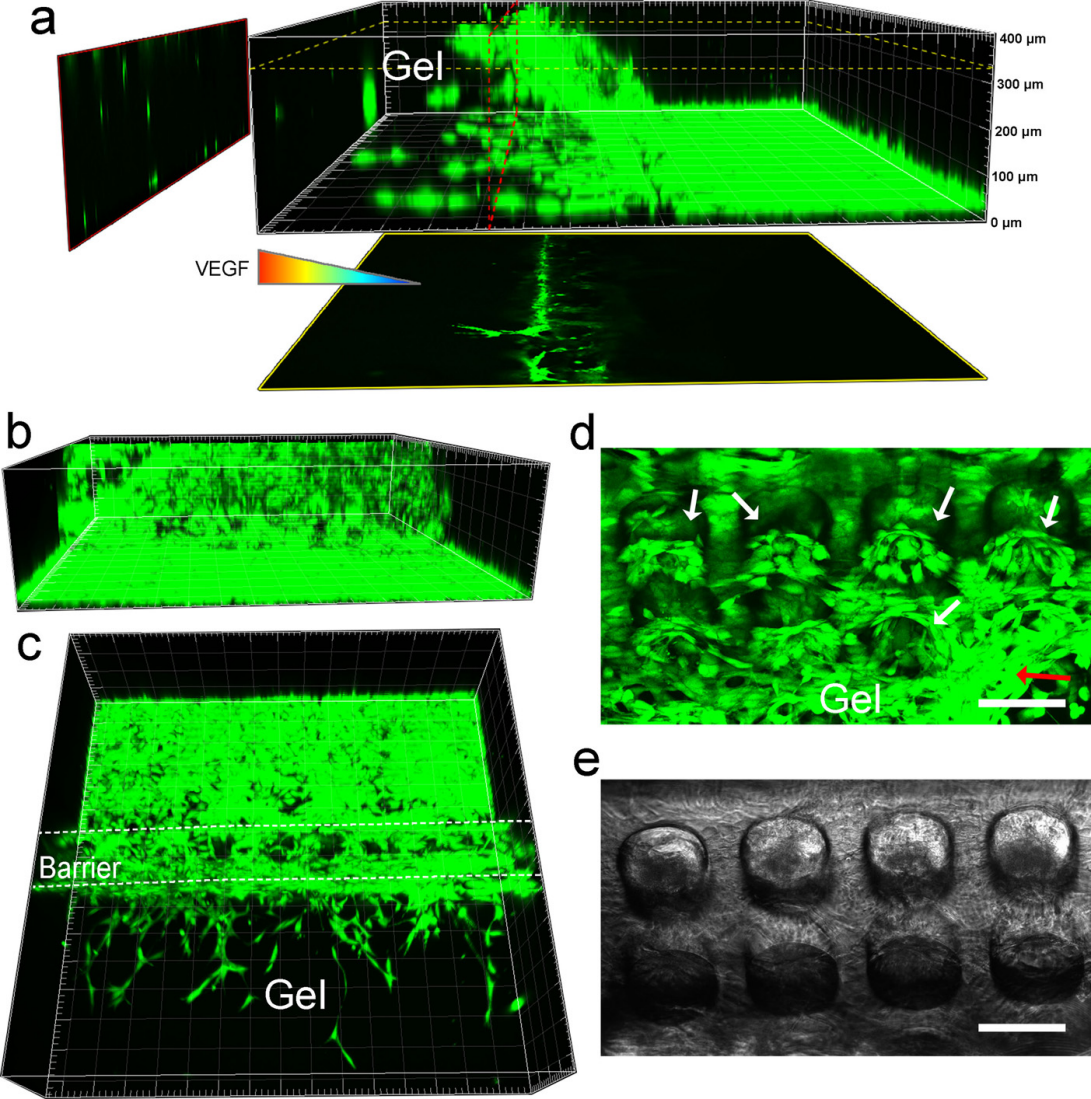
Three-dimensional observation of cell sprouting. (a) Lateral view of cells sprouting on the barrier after 3 days of culture. A horizontal sectional view is shown in the yellow frame, and a vertical sectional view is shown in the red frame. (b) and (c) The back view and top view of (a), respectively. (d) and (e) The fluorescence micrograph and phase-contrast micrograph of cells after 3 days of culture. The white arrows indicate the annular distribution of cells. The red arrow indicates the cell sprouting from a microhole. The scale bar is 100 *μ*m.

Since the HUVECs had formed several, but not very dense, sprouts in the device with the barrier after 3 days of culture, it was suitable for observing the growth positions of the sprouts by 3D reconstruction of the fluorescence images. A lateral view of the cells growing on the barrier [Fig. 7(a)] showed that the cells sprouted from different heights of the barrier into the collagen scaffold and that the sprouts closer to the bottom of gel scaffold grew longer. A horizontal sectional view of the sprouts at a 330–*μ*m distance from the bottom [yellow frame in Fig. 7(a)] showed that one sprout had migrated approximately 200 *μ*m into the 3D gel scaffold. The vertical sectional view [red frame in Fig. 7(a)] showed that there were several sprouts in the middle-upper region and more sprouts in the near-bottom region. There are two potential reasons why there were more sprouts near the bottom and why they were longer: (1) In the cell chamber, many cells grow on the glass substrate. Therefore, many cells will migrate into the near-bottom region of the collagen. (2) Cells tend to migrate to stiffer regions, which is known as “durotaxis.”^43–45^ Due to the presence of the glass substrate, the near-bottom region of the collagen provided a stiffer environment for the cells.^46^ Figures 7(b) and 7(c) show the back view and top view of Fig. 7(a), respectively. The results show that the barrier had been full of cells [Fig. 7(b)] and that many sprouts grew out of the barrier [Fig. 7(c)]. The resolution of the cells in the microholes was poor in the 3D reconstruction; therefore, we tilted the barrier to investigate the details of cell sprouting in the microhole region [Figs. 7(d) and 7(e)]. The results showed that HUVECs had migrated out of the microhole and formed an annular distribution [indicated by the white arrows in Fig. 7(d)].^27,41^ This is considered to be conducive for the formation of more regular sprouts.^20,29^ Furthermore, some of the cells had sprouted into the gel scaffold [indicated by the red arrow in Fig. 7(d)].

The analysis of the above results suggest that the process of EC sprouting in the microhole barrier is as follows: ECs attach to the surface of the microhole barrier and form an annular distribution in the inner wall of the microhole; ECs then migrate into the gel scaffold and form regularly distributed sprouts; and with the increasing lengths of the sprouts, they gradually form many well-interconnected tube-like structures and eventually have the potential to form a microvascular network.^13,29^ In contrast to the dense and disordered sprouting when ECs were cultured without the barrier, the sprouts in the gel scaffold with the microhole barrier were more consistent with the complex vascular distribution that is observed *in vivo*^20,21,47^ and is, thus, an ideal *in vitro* angiogenesis model.

## IV. CONCLUSIONS

In this study, we developed a PDMS microfluidic device using the coverslip molding method, which was convenient and inexpensive. We were able to establish a stable VEGF concentration gradient, which induced HUVECs to migrate and sprout into the collagen scaffold. We also assembled a unique PDMS microhole barrier at the boundary of the collagen. HUVECs cultured on the microhole barrier formed longer and more regular sprouts than HUVECs cultured on the control device (without barrier) and could form more well-interconnected tube-like structures, which were more consistent with the distribution of microvasculature *in vivo*. The integration of the microtopographic structure and 3D collagen in the microfluidic device is able to bring out the advantages of both features for 3D cell culture. In summary, this novel microfluidic device is an ideal platform for angiogenesis research and drug screening *in vitro*.

## V. SUPPLEMENTARY MATERIAL

See supplementary material for the simulation of VEGF diffusion, detailed statistical methods of HUVEC sprouting, and additional experimental data.
